# MOCCASIN: converting MATLAB ODE models to SBML

**DOI:** 10.1093/bioinformatics/btw056

**Published:** 2016-02-09

**Authors:** Harold F. Gómez, Michael Hucka, Sarah M. Keating, German Nudelman, Dagmar Iber, Stuart C. Sealfon

**Affiliations:** ^1^Department of Biosystems Science and Engineering, ETH Zürich, Basel CH 4058, Switzerland,; ^2^Computing and Mathematical Sciences, California Institute of Technology, Pasadena, CA 91125, USA,; ^3^European Molecular Biology Laboratory, European Bioinformatics Institute (EMBL-EBI), Hinxton, Cambridge CB10 1SD, UK and; ^4^Department of Neurology, Icahn School of Medicine at Mount Sinai, Mount Sinai Medical Center and School of Medicine, New York, NY 10029, USA

## Abstract

**Summary:** MATLAB is popular in biological research for creating and simulating models that use ordinary differential equations (ODEs). However, sharing or using these models outside of MATLAB is often problematic. A community standard such as Systems Biology Markup Language (SBML) can serve as a neutral exchange format, but translating models from MATLAB to SBML can be challenging—especially for legacy models not written with translation in mind. We developed MOCCASIN (*Model ODE Converter for Creating Automated SBML INteroperability*) to help. MOCCASIN can convert ODE-based MATLAB models of biochemical reaction networks into the SBML format.

**Availability and implementation:** MOCCASIN is available under the terms of the LGPL 2.1 license (http://www.gnu.org/licenses/lgpl-2.1.html). Source code, binaries and test cases can be freely obtained from https://github.com/sbmlteam/moccasin.

**Contact**: mhucka@caltech.edu

**Supplementary information:** More information is available at https://github.com/sbmlteam/moccasin.

## 1 Introduction

MATLAB is a general-purpose numerical computing environment whose powerful features have attracted many researchers. It has been the substrate for countless models as well as software tools written in its object-oriented programming language. Despite its popularity, there are reasons why MATLAB programs are not themselves a desirable format for exchanging, publishing or archiving computational models in biology. These include the lack of biological semantics in MATLAB programs, which makes clear interpretation of programs as models of biological processes more difficult; the fact that MATLAB is proprietary and expensive, which makes it unsuitable as a universal format for open scientific exchange; and the fact that model details are often intertwined with program implementation details, which makes it difficult to determine which parts constitute the essence of a model.

Systems Biology Markup Language (SBML) is an open format for representing models in systems biology (Hucka *et al.*, 2003). Designed to resolve incompatibilities between systems that use different formats to describe models, SBML is neutral with respect to modeling framework and computational platform. This helps make models portable across tools, and ensures that models as research products can persist regardless of changes to any particular software tool or operating system. Unfortunately, translating models from MATLAB to SBML is not straightforward. Some MATLAB toolboxes (Keating *et al.*, 2006; Schmidt and Mats, 2006) offer SBML capabilities; however, they have limited utility for translating legacy models, lack support for the latest SBML releases, and must be used from the start of a modeling project to have an effect.

These issues led us to develop Model ODE Converter for Creating Automated SBML Interoperability (MOCCASIN), a stand-alone tool that can take ODE models written in MATLAB and export them as SBML files. MOCCASIN is written in Python and does not require access to MATLAB. To develop it, we drew on recent advances in the inference of biochemical reaction networks (Fages *et al.*, 2015). The result allows for richer SBML that can also be used for qualitative analyses where knowledge of the reaction network behind a system of ODEs is required.

## 2 Implementation

MOCCASIN features a modular architecture comprised of (i) a module that parses MATLAB files; (ii) a module that extracts the ODE-based model and produces a model with explicit ODEs; (iii) a module that infers the biochemical reactions implied by the ODEs and produces SBML output with biochemical reactions for kinetics; (iv) a command line interface and (v) a graphical user interface. Python developers can use as few or as many modules as they desire.

### 2.1 Parsing module

MATLAB is difficult to parse fully (Doherty *et al.*, 2011): the language is complex and idiosyncratic, and there is no published definition of its syntax rules. We did not attempt to develop a complete parser for MATLAB; instead, we leveraged the fact that MOCCASIN's input is already expected to be syntactically valid MATLAB (because users are converting working code), and thus MOCCASIN's parser can be simpler and make more assumptions. The parser creates an internal representation that is essentially an embellished Abstract Syntax Tree (AST).

### 2.2 Converter module

The AST is processed to recognize specific constructs. The approach centers on finding a call to one of the MATLAB ode*NN* family of solvers (e.g. ode45, ode15s, etc.). Once this is found, the converter inspects the AST for the definitions of the arguments to the call; these are expected to be either a matrix or the handle of a function that returns a matrix. If it is a function (which must be found elsewhere in the same file), MOCCASIN inspects the parsed function body. The rows of the matrix or the function's return values are assumed to define the ODEs of the user's model. MOCCASIN translates this and generates either an SBML (using SBML ‘rate rules’) or XPP (Ermentrout, 2002) representation. For SBML, MOCCASIN makes use of libSBML (Bornstein *et al.*, 2008); to generate XPP, it directly implements the necessary translation.

### 2.3 BIOCHAM module

Encoding a model's ODE equations in a one-to-one fashion using SBML's ‘rate equations’ is sufficient to ensure simulation reproducibility, but the translated model is not ideal if the original system of ODEs actually represents a biochemical reaction network. Reconstructing this network captures the underlying model more productively and enables subsequent application of analyses that require biochemical reactions (Gay *et al.*, 2014). To export SBML models with fully resolved reaction networks, MOCCASIN sends the converter's output via web services to BIOCHAM, a modeling environment that incorporates a state-of-the-art algorithm for reconstructing and inferring the complete reaction model from a given set of ODEs (Fages *et al.*, 2015). Due limitations in the XPP format and the BIOCHAM service, the result lacks some components present in the original model. MOCCASIN therefore post-processes the output from BIOCHAM to add initial assignments, references to the time variable (if used in the original model) and other pieces. All components of the initial MATLAB ODE model are thus captured, and each reaction in the SBML output is fully characterized with well-identified reactants, products and modifiers.

### 2.4 Command-line interface

MOCCASIN provides a cross-platform command-line interface (CLI) that facilitates scripting and automation.

### 2.5 Graphical user interface

The GUI interface is implemented with a cross-platform GUI toolkit. The interface provides a straightforward way for users to input MATLAB files, set MOCCASIN options such as the type of output (SBML or XPP), view the resulting output and save the converted file.

## Future work

The MATLAB input currently must conform to certain simple forms and make limited use of MATLAB features. Future enhancements will (i) expand the set of MATLAB constructs that can be interpreted; (ii) support models spread over several MATLAB input files; (iii) generate SED-ML (Simulation Experiment Description Markup Language; Waltemath *et al.*, 2011) files, to encode procedural aspects that cannot be expressed in SBML and (iv) directly implement the BIOCHAM reaction inference algorithm (Fages *et al.*, 2015), to streamline the translation process.
